# Prevalence of Colistin-Resistant *Klebsiella pneumoniae* Isolates in Turkey over a 20-Year Period: A Systematic Review and Meta-Analysis

**DOI:** 10.3390/microorganisms13050974

**Published:** 2025-04-24

**Authors:** Elmas Pinar Kahraman Kilbas, Imdat Kilbas, Ihsan Hakki Ciftci

**Affiliations:** 1 Department of Medical Laboratory Techniques, Health Services Vocational School, Fenerbahce University, 34758 Istanbul, Turkey; elmspnrkk@gmail.com; 2 Medical Microbiology Doctorate Program, Institute of Health Sciences, Istanbul University, 34093 Istanbul, Turkey; imdtklbs@gmail.com; 3 Department of Medical Microbiology, Faculty of Medicine, Sakarya University, 54100 Sakarya, Turkey

**Keywords:** healthcare-associated infections, multidrug resistance, Gram-negative bacteria, epidemiology

## Abstract

*Klebsiella pneumoniae* is one of the leading causes of healthcare-associated infections and poses challenges in its treatment owing to its high antibiotic resistance. The development of resistance to colistin, which is used as a last resort, has become a major public health problem worldwide. This study was planned according to the PRISMA guidelines and included studies reporting the prevalence of colistin-resistant *K. pneumoniae* in Turkey between 2004 and 2024 through a systematic literature review. A total of 28 original research articles were included in the meta-analysis. Data were analyzed using the SPSS and CMA software. The pooled colistin resistance of a total of 8916 *K. pneumoniae* strains from 28 studies included in this meta-analysis was found to be 1.63% (95% CI: 1.51–3.12). Colistin resistance increased significantly over time. A higher resistance rate was detected in the strains tested using the EUCAST guidelines and broth microdilution method. The year of the study and validation methods contributed to the heterogeneity observed in the studies. This meta-analysis reveals that colistin-resistant *K. pneumoniae* strains have increased over time in Turkey. Current data show that colistin resistance is not only a laboratory finding but has become a crisis, requiring urgent action in terms of hospital infection management and patient safety. Regional and global measures should be taken to ensure the appropriate use of antibiotics to control the development of resistance.

## 1. Introduction

*Klebsiella pneumoniae* is a leading cause of healthcare-associated infections worldwide and is a member of the ESKAPE group (*Enterococcus faecium*, *Staphylococcus aureus*, *K. pneumoniae*, *Acinetobacter baumannii*, *Pseudomonas aeruginosa* and *Enterobacter* spp.), the majority of which are resistant to antimicrobial agents [1]. *K. pneumoniae* has an important place among healthcare-associated infectious agents and causes infections such as pneumonia, urinary tract infections, septicemia and meningitis. It is an important public health problem that causes critical morbidity and mortality, particularly in hospitalized patients [2]. Immunocompromised patients, those in intensive care units, those with malignancies and those using urinary catheters are among the groups at the highest risk for *K. pneumoniae* infections [3]. Many Gram-negative bacteria cause nosocomial infections; however, *K. pneumoniae* stands out because of its high antibiotic resistance [4,5]. Carbapenemases such as *bla*_KPC_ (*K. pneumoniae* carbapenemase) and *bla*_NDM_ (New Delhi metallo-beta-lactamase), which have been widely reported in recent years, have rendered the treatment of *K. pneumoniae* significantly difficult due to antimicrobial resistance [6].

The overuse of antibiotics has led to problems in the treatment of infections caused by *K. pneumoniae*. Therefore, colistin has been used as a last-resort treatment [7]. Colistin has been reintroduced because of its activity against Gram-negative bacteria that have developed resistance to existing antibiotics, but issues such as nephrotoxicity and neurotoxicity remain open to debate [8]. Colistin, also known as polymyxin E, is a last-resort antibiotic against multidrug-resistant infections caused by Gram-negative bacteria such as Enterobacteriaceae [9]. Polymyxins are cationic antimicrobial peptides that target the phosphate moiety of bacterial lipopolysaccharide (LPS), disrupt the negative charge of the outer membrane and cause cell death [10]. However, derivatives such as polymyxin B and polymyxin E, developed to reduce nephrotoxicity and nephrotoxic effects, offer different options in clinical use [11]. However, in recent years, many studies have reported the detection of colistin-resistant *K. pneumoniae* isolates worldwide [12,13]. The colistin resistance rate in *K. pneumoniae* strains worldwide was reported as 12.90% in isolates examined in 2020 and later [14]. The prevalence of this resistance necessitates the more stringent implementation of infection control measures, particularly for healthcare-associated infections.

In addition, it has been shown that colistin resistance is selected together with the use of broad-spectrum cephalosporins in *K. pneumoniae* [15]. In this process, the selective pressure caused by broad-spectrum cephalosporins triggers the development of colistin resistance by causing mutations in the chromosomal *mgrB* gene via transposition. These findings show that the inappropriate use of different groups of antibiotics may also increase the resistance to critical antibiotics such as colistin.

Various treatment methods, such as phage therapy, herbal medicines, photodynamic therapy, antimicrobial peptides and nanoantibiotics, are being investigated for the treatment of infections caused by these resistant strains. In particular, recently developed nanoformulations and metal-based nanoparticles offer promising alternatives against resistant bacteria such as *K. pneumoniae* [16].

To date, colistin resistance has been thought to develop through chromosome or horizontal gene transfer [17]. However, mutations in two-component systems, including PmrA/PmrB and PhoP/PhoQ, and alterations in the *mgrB* gene, which encodes the negative regulator of PhoPQ, have been identified as the main causes of colistin resistance in *K. pneumoniae* [18]. Bioinformatics analyses performed in recent years have shown that these mutations in *K. pneumoniae* colistin resistance genes have become widespread in hospital-acquired strains [19].

In addition, plasmid-mediated colistin resistance genes associated with colistin resistance have been reported in some countries in recent years [12,13]. The *mcr-1* gene region has spread horizontally among bacteria across most continents. It has been demonstrated in bacteria isolated from foods, environmental samples, patients with infections and asymptomatic international travelers [20]. After *mcr-1*, the plasmid-mediated colistin resistance gene was first identified in late 2015, and nine new *mcr* variants have been reported to date, namely *mcr-2*, *mcr-3*, *mcr-4*, *mcr-5*, *mcr-6*, *mcr-7*, *mcr8*, *mcr-9* and the recently identified *mcr-10* [21]. There are few studies on the genes responsible for colistin resistance in *K. pneumoniae* isolates in Turkey. In some studies conducted in our country, *mcr-1* positivity was reported in isolates isolated from clinical samples [22,23].

This study aimed to compare the prevalence rates reported in studies examining the prevalence of colistin-resistant *K. pneumoniae* isolates in Turkey between 2004 and 2024 in terms of different parameters, such as the geographical region and diagnostic methods used.

## 2. Materials and Methods

### 2.1. Protocol

This study was planned based on the Preferred Reporting Items for Systematic Reviews and Meta-Analyses (PRISMA) procedure guidelines [24]. This systematic review and meta-analysis study was registered with PROSPERO with the number “CRD420251000119”.

### 2.2. Literature Search

A systematic search was conducted using different electronic databases, including PubMed, Embase, Web of Science, EBSCO, Scopus, Turk Medline and Google Scholar, between January 2004 and February 2024. The search was performed in the English and Turkish languages using keywords such as “prevalence of colistin resistance of *Klebsiella pneumoniae* isolates in Turkey”, “kolistin dirençli *Klebsiella pneumoniae*”, “colistin-resistant *Klebsiella pneumoniae*”, “kolistine dirençli *Klebsiella pneumoniae* prevalansı” and “prevalence of colistin-resistant *Klebsiella pneumoniae*”. The same search strategy, including keywords, was applied across all databases to ensure consistency.

### 2.3. Exclusion Criteria

Studies whose full texts could not be accessed or did not provide a clear number, studies with inconsistent data (studies that did not clearly report the number of strains and resistance rates), studies conducted outside of clinical samples, studies that did not provide species-level definitions, theses, congress presentations, compilations, systematic reviews and case report studies were not included in this study. To reduce heterogeneity, articles describing fewer than 10 strains were also not included in this study.

### 2.4. Inclusion Criteria

Original/research articles published in national and international peer-reviewed journals, studies whose full texts were accessible in databases, studies with species-level definitions, studies conducted with clinical samples, articles reporting the prevalence of colistin-resistant *K. pneumoniae* clinical isolates using standard laboratory tests, studies published between January 2004 and December 2024, those conducted in Turkey and those published in Turkish or English were included in the meta-analysis. The quality of the included studies was assessed using the critical appraisal checklist designed by the Joanna Briggs Institute (JBI). A “Yes” answer to nine questions in the checklist was given 1 point. The total score for each study ranged from zero to nine. A study with a score of 4–6 was considered of medium quality, and a study with a score of 7–9 was considered of high quality [25]. All included studies had scores above 5. According to this assessment, 18 studies were of medium quality, and 10 studies were of high quality.

### 2.5. Data Evaluation and Statistical Analysis

**Figure 1:**
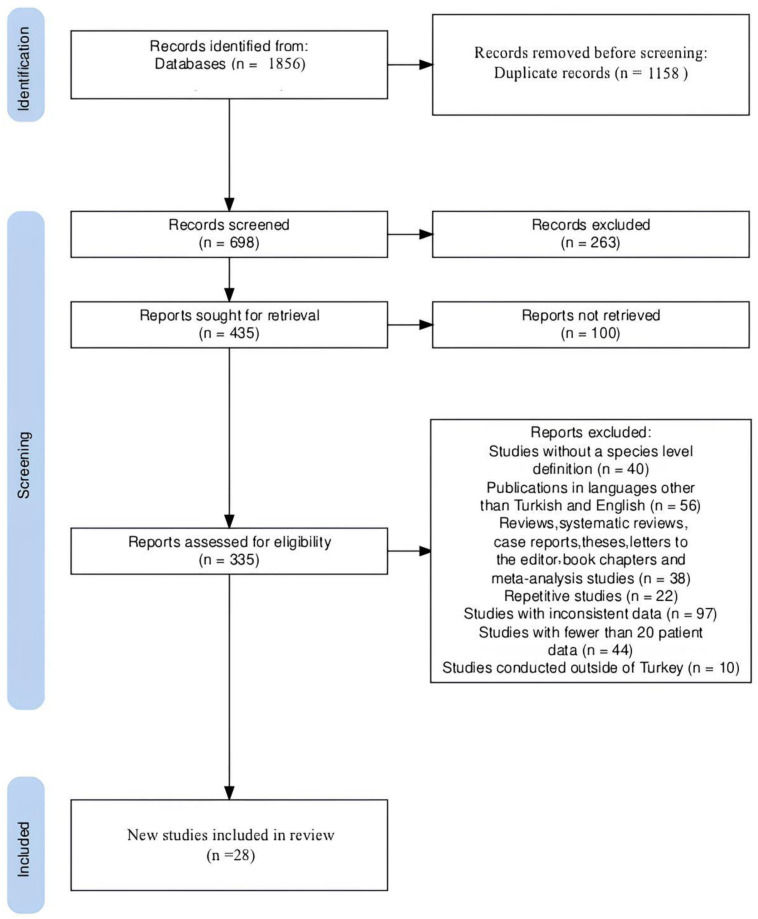
Studies were excluded and included based on the above search criteria (PRISMA flowchart).

Abstracts and full texts were independently searched by three researchers, according to the eligibility criteria for data extraction. The results were reviewed by the relevant authors, and differences between the researchers were resolved through consensus and discussion. The data extraction form for the included articles included the first author, year of publication, city where the study was conducted, total number of isolates, number of colistin-resistant isolates, diagnostic methods, methods used to confirm colistin resistance and resistance detection guidelines (Supplementary Table S1). The total number of studies found according to the keywords applied in the databases was 1856, and the full texts of 435 studies were accessed. A total of 28 original research articles were included in this study after being evaluated according to the exclusion criteria (Figure 1) [22,23,26,27,28,29,30,31,32,33,34,35,36,37,38,39,40,41,42,43,44,45,46,47,48,49,50,51].

The data were analyzed using the SPSS software (IBM SPSS Statistics, Version 25.0; IBM Corp., Armonk, NY, USA). Independent-sample *t*-tests and one-way ANOVA tests were used to compare continuous data.

A random-effects model was used for the meta-analysis. Statistical significance was set at *p* < 0.05, with 95% confidence intervals. The heterogeneity level was determined by I-square (i^2^) statistics. The Egger test was performed using the SPSS software to assess publication bias.

To determine possible factors of heterogeneity, the relationship between the study year, region, antimicrobial resistance detection method and effect size was evaluated by meta-regression analysis with the Comprehensive Meta-Analysis (CMA) software, ver. 3.3 (Biostat, Englewood, CO, USA). The extent to which the factors explaining the reasons for the heterogeneity explained the variability in the effect size was revealed by the R-squared (R^2^) value. In the analyses, the effect of subgroups was tested statistically.

## 3. Results

A total of 8916 *K. pneumoniae* strains from 28 different research articles were included in this meta-analysis. Of these strains, 1197 (13.43%) were resistant to colistin. The pooled prevalence of colistin resistance was calculated as 1.63% (95% CI: 1.51–3.12). The studies were divided into three time periods (2004–2013, 2014–2018, 2019–2024) to better assess changes in antibiotic resistance. This separation allowed us to observe the effects of health policies such as the “Rational Use of Antibiotics”, implemented in Turkey in 2014, and the changes in the resistance rates due to antibiotic use caused by the COVID-19 pandemic after 2019. It was observed that colistin resistance showed a statistically significant increase over time (*p* < 0.05; *p* = 0.00).

The most frequently used devices for the identification of *K. pneumoniae* strains were the VITEK-2 (bioMérieux, Marcy-l’Étoile, France) (13/28), MALDI-TOF MS (Bruker Daltonics, Billerica, MA, USA) (5/28), and the BD Phoenix (Becton Dickinson, Franklin Lakes, NJ, USA) (5/28). The EUCAST (19/28), CLSI (6/28), and EUCAST-CLSI guidelines (2/28) were used most frequently for antimicrobial resistance testing, and the guidelines used were not specified in one study. It was observed that the resistance rates detected in studies using only the EUCAST guidelines and in studies using both guidelines together were higher compared to studies using the CLSI guidelines (*p* < 0.05; *p* = 0.02) (Supplementary Table S1). Broth microdilution (10 studies), Kirby–Bauer disk diffusion (seven studies), and gradient strip tests (four studies) were the most frequently used tests to confirm colistin resistance. It was determined that 551 of 2553 strains (21.58%) included in the studies conducted with the broth microdilution test and 175 of 4253 strains (3.87%) included in the studies conducted with the Kirby–Bauer disk diffusion test were colistin-resistant. The pooled colistin resistance prevalence reported in the studies using the broth microdilution method was found to be 15.70% (95% CI: 14.76–16.65).

Although different samples were used together in the studies, the most frequently used types of samples were blood (20 studies), urine (15 studies), tracheal aspirates (11 studies), wound samples (eight studies), and sputum (six studies).

In our study, the average colistin resistance rates of *K. pneumoniae* strains isolated from different geographical regions were 34.83% in the Black Sea region, 33.32% in the Southeastern Anatolia region, 29.30% in the Marmara region, and 28.04% in the Central Anatolia region (*p* > 0.05; *p* = 0.49). Since only one study was included from the Mediterranean, Aegean, and Eastern Anatolia regions, the average could not be calculated for these regions.

The *mcr-1* gene was reported at rates of 1.75%, 0%, 7.44%, 48.5%, and 0% in the studies of Arabacı et al. (2019), Özkaya et al. (2020), Kansak et al. (2020), Genişel et al. (2021), and Suzuk Yildiz et al. (2021), respectively [22,23,36,41,44]. The reason for the high rate in the study of Genişel et al. (2021) was that all strains were isolated from patients in the intensive care unit [41]. The *mcr-8* gene was investigated only in the study of Suzuk Yildiz et al. (2021), and it was reported that this gene region was not found in any strain [44].

### Publication Bias and Heterogeneity

Egger’s regression test was applied to assess publication bias. The results showed no statistically significant evidence of publication bias (t = −0.621, *p* = 0.540; *p* > 0.05).

According to the random-effects model, the average effect size of the 28 studies included in the meta-analysis was calculated as 0.23, with an upper limit of 0.328 and a lower limit of 0.155 in the 95% confidence interval (Z = −4.843; *p* < 0.001). According to Cohen et al. [52], this effect size is medium.

**Figure 2:**
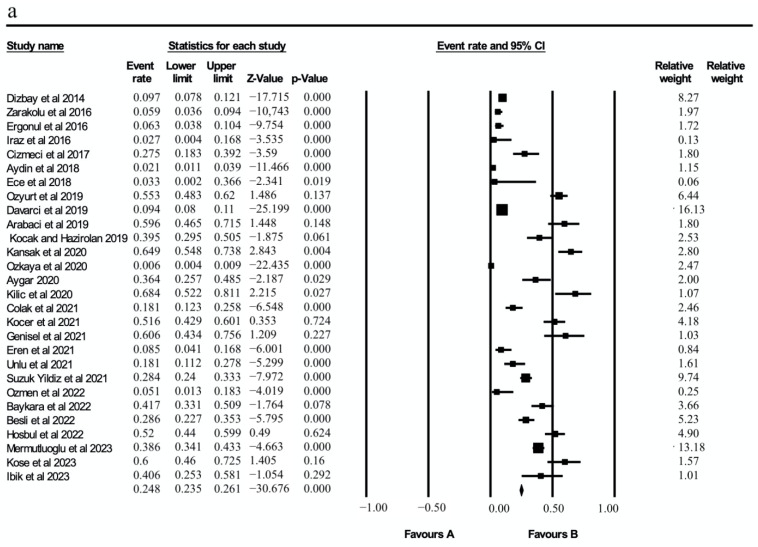

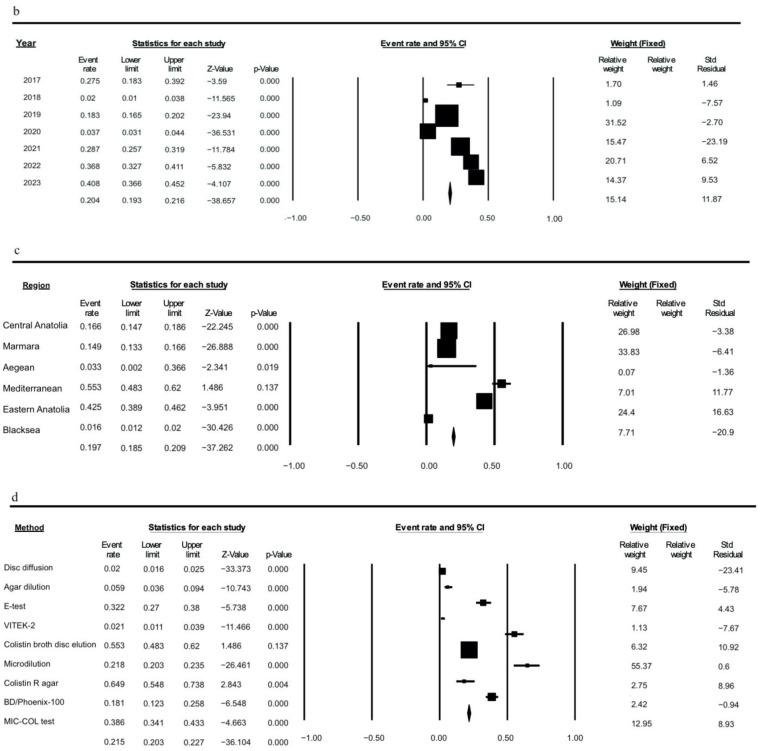
(**a**) General colistin resistance determined in studies, (**b**) colistin resistance rates by year, (**c**) colistin resistance rates by region and (**d**) forest plot analysis of colistin resistance rates by method [22,23,26,27,28,29,30,31,32,33,34,35,36,37,38,39,40,41,42,43,44,45,46,47,48,49,50,51].

The data for each result were summarized in a forest plot graph. The effect sizes of the studies had high heterogeneity (i^2^ = 97.67%). This high heterogeneity may have been due to the fact that the studies were conducted in different regions and with different methodologies. It can be stated that the confidence intervals of most of the studies were narrow, and the data provided reliable results (Figure 2a). In the graph, it is seen that the confidence intervals of some studies were wide [31,41,51]. This was due to the small sample sizes of these studies. The effect sizes of the colistin resistance rates by year, region and method also showed high heterogeneity (i^2^ = 99.2, 99.35, 99.07%) (Figure 2b–d).

The meta-regression analysis, which was performed to determine the factors affecting heterogeneity, was performed via the logit event rate using a random-effects model. The geographical region did not have a significant effect on the prevalence of colistin resistance (*p* > 0.05; *p* = 0.45). The variance that the model could not explain was high (i^2^ = 97.77%), and there was high heterogeneity among the studies. These findings suggest that the differences in colistin resistance prevalence may have been due to factors other than the geographical region (R^2^ analog = 0.00). It was observed that the confirmation method had a statistically significant effect on the colistin resistance prevalence (β = 0.2687, *p* < 0.05; *p* = 0.00). A positive coefficient indicated that the prevalence of colistin resistance may increase with the use of resistance confirmation methods. The coefficient of the year variable was found to be statistically significant (β = 0.0006, *p* < 0.05; *p* = 0.00). This finding indicates that there has been an increasing trend in the prevalence of colistin resistance over the years. The year and confirmation method variable exhibited a significant contribution in explaining the heterogeneity in the prevalence of colistin resistance.

## 4. Discussion

In 2021, 1.14 million deaths were directly attributed to bacterial antimicrobial resistance. While the mortality rates in children under 5 years of age have decreased over the last 31 years, the mortality rates in adults aged 70 years and over have increased by more than 80%. Deaths due to antimicrobial resistance are expected to increase to 8.22 million in 2050, and it has been reported that approximately 103 million deaths could be prevented with resistance control strategies [53]. In 2021, *K. pneumoniae* was among the top four pathogens causing death due to antimicrobial resistance [54]. *K. pneumoniae* is an opportunistic pathogen responsible for approximately 10% of hospital-acquired bacterial infections.

This meta-analysis highlights the threat to public health by revealing the prevalence of *K. pneumoniae* isolates that are resistant to colistin, which is used as a last resort against MDR bacteria. Infections caused by colistin-resistant *K. pneumoniae* isolates, particularly in intensive care patients, pose a major threat to public health [55]. As these infections increase healthcare costs, prolong the length of stay in intensive care units and increase the mortality rate among patients, their treatment is of great importance [56]. Colistin resistance has become a concern due to mutations in chromosomal genes and plasmid-mediated colistin resistance mechanisms in *K. pneumoniae* strains. Factors that trigger an increase in colistin resistance include the unnecessary use of antibiotics, a lack of hygiene standards and the spread of resistant strains. A more detailed examination of these factors will contribute to the development of strategies to combat resistance [57]. This meta-analysis revealed the prevalence of colistin resistance in *K. pneumoniae* strains isolated from patients treated at various clinics throughout Turkey.

Although the large molecular structure of colistin and its binding properties to plastic surfaces negatively affect the reliability of disk diffusion tests, the broth microdilution test is the most reliable method in accurately determining antimicrobial susceptibility [58]. This study shows that the broth microdilution test was used in the majority of the included studies and that this test detected higher rates of colistin resistance. A total of 1197 (13.43%) of the 8916 *K. pneumoniae* strains in the studies included in the meta-analysis were found to be resistant to colistin. A total of 551 (21.58%) of the 2553 strains included in the studies performed with the broth microdilution test and 175 (3.87%) of the 4253 strains included in the studies performed with the Kirby–Bauer disk diffusion test were found to be resistant to colistin. The pooled colistin resistance rate was 1.63%, and the prevalence of pooled colistin resistance reported in studies using the broth microdilution method was 15.70%. These findings reveal the critical role that the testing methods play in determining the resistance rates. Therefore, adopting broth microdilution testing as a routine practice to confirm colistin resistance in clinical laboratories is important to determine the true prevalence of colistin resistance.

In a study by Poonam et al. [59], one of 17 *K. pneumoniae* strains was found to be resistant via the disk diffusion method, while six isolates were found to be resistant to colistin via the broth microdilution method. The broth microdilution test is the gold-standard method for the reliable determination of colistin resistance. Considering the limitations of the disk diffusion test, it is critical to include broth microdilution testing in routine diagnostic processes, particularly in regions where colistin resistance is highly prevalent. Uzairue et al. [14] found the pooled colistin resistance rate to be 3.1% in their meta-analysis, which included studies that examined colistin resistance in *K. pneumoniae* strains using a broth microdilution test. Narimisa et al. [3] reported a pooled colistin resistance prevalence of 6.9% in meta-analysis studies in Iran, where colistin resistance was tested using the broth microdilution and disk diffusion methods. While the colistin resistance rate in *K. pneumoniae* strains was reported as 26.9% in Greece in 2016, the colistin resistance rate was found to be 73.9% in a different study conducted in Greece between 2014 and 2017. In both studies, the EUCAST standards were applied and colistin resistance was confirmed by the broth microdilution method [60,61]. In a study conducted in Bulgaria between 2017 and 2018, a colistin resistance rate of 26% was reported [62]. These different rates between Turkey and neighboring countries can be explained by the effects of antibiotic use policies, especially in the treatment of resistant bacterial infections. These differences also reveal that the hygiene practices and public health policies may also be different. Our data, which reveal the resistance rates in Turkey, especially as a result of comparisons performed with meta-analyses, show that we have lower rates compared to others in the fight against colistin resistance, considering the regional characteristics and clinical conditions.

Since 2015, the EUCAST standards have been used for antimicrobial susceptibility testing in Turkey [63]. The main difference between EUCAST and CLSI is the reduction or removal of the intermediate category. EUCAST partially removed the intermediate region owing to pharmacodynamic–pharmacokinetic data and limited clinical evidence and classified AST reports as susceptible or resistant. Therefore, when the antibiotic susceptibilities of bacteria were evaluated according to the EUCAST guidelines, Gram-negative microorganisms were found to be more resistant to antimicrobials [64]. The EUCAST guidelines were used to determine antibiotic susceptibility in most studies included in this meta-analysis. In these studies, it was observed that the resistance rates detected in those using the EUCAST guidelines and in those using both the CLSI and EUCAST guidelines were higher compared to the studies using only the CLSI guidelines. The broth microdilution method was used to confirm colistin resistance in 9 of the 19 studies using the EUCAST guidelines. The guidelines used in the evaluation of antimicrobial susceptibility have led to significant differences in the evaluation of the bacterial resistance rates. This situation again reveals the effects of the methodology and standards used on the reported resistance rates [65].

The colistin resistance in the studies included in the meta-analysis increased over time. In particular, the two-fold increase in the colistin resistance rate between 2019 and 2024 is striking. This increase may be related to the use of colistin, which was frequently used to treat infections caused by carbapenem-resistant *K. pneumoniae* during the COVID-19 pandemic in 2020. Significant changes were observed in the methods used in colistin susceptibility testing during the three time periods covered in the study (2004–2013, 2014–2018, 2019–2024). While diffusion methods were mostly preferred to confirm colistin resistance between 2004 and 2013, diffusion and dilution methods were used equally between 2014 and 2018. In the 2019–2024 period, the use of reference methods such as broth microdilution increased significantly. In particular, broth microdilution is known to yield higher resistance rates compared to other methods. Therefore, it should be kept in mind that the resistance changes observed in this study may be due not only to epidemiological trends but also to differences in the test methods used. In the study conducted by Kurt et al. [66] to investigate the effects of the COVID-19 pandemic on antibiotic resistance in *K. pneumoniae* strains, it was stated that colistin resistance increased from 50.5% in the pre-pandemic period to 61.7% in the post-pandemic period. The increase in antibiotic resistance observed in Gram-negative bacteria after the COVID-19 pandemic may be due to the disruption of infection control measures in hospitals during this period. The COVID-19 pandemic has affected antimicrobial resistance rates through changes in infection control measures and antibiotic use. A study conducted in South Korea showed that the pandemic was associated with changes in the prevalence of allergic rhinitis and chronic rhinosinusitis [67]. Similarly, in our study, it was observed that the antibiotic resistance rates in Klebsiella strains changed during the pandemic period. While the increased use of broad-spectrum antibiotics due to the pandemic may have accelerated the development of resistance, infection control measures may have caused different trends in the resistance rates to certain antibiotics. Considering that colistin is the last-choice antibiotic, it has become vital in recent years for healthcare workers to implement infection control measures, such as hand washing and glove use, to control the spread of these strains. The changes observed during this period require further studies to better understand the effects of the pandemic on antibiotic resistance.

Only six of the studies included in the meta-analysis investigated *mcr* genes in *K. pneumoniae* strains. The prevalence rates ranged from 0% to 48.5%. The variability in the rates may vary depending on factors such as the patient group studied, clinical setting, immunosuppression, geographic region, hospital type and methodologies used. Recent studies on antibiotic resistance have shown that pyridoxal 5′-phosphate (PLP), the active form of vitamin B6, is significantly reduced in colistin-resistant *mcr*-positive bacteria. Therefore, it has been suggested that PLP supplementation may restore colistin’s efficacy by reversing the metabolic profile of resistant bacteria [68]. These findings suggest that metabolically directed antibiotic sensitization strategies may offer a new approach against antibiotic resistance. In addition to these metabolic strategies, the integration of advanced technologies can further accelerate the identification of resistant strains, improving treatment outcomes. Following the COVID-19 pandemic, patients can be identified quickly thanks to robotic systems and deep learning-supported patient assessment technologies developed in recent years [69]. Similarly, these technologies can help to identify antimicrobial-resistant strains more quickly. For example, various image analysis techniques can help to identify resistant strains more quickly and reliably by analyzing microbiological data from patient samples.

In this meta-analysis, publication bias was not detected in a statistically significant manner, which increases the reliability of the findings. The moderate effect size of the studies showed that the data on colistin resistance were consistent and evaluable. The high heterogeneity (97.67%) may be related to factors such as the geographical regions in which the studies were conducted, study methodology, patient profiles and antibiotic susceptibility test evaluation guidelines. The small sample sizes of the studies with wide confidence intervals in the forest plot analysis may also be one of the factors increasing the heterogeneity.

As a result of the meta-regression analysis, the significant effect of colistin resistance confirmation methods on the prevalence of resistance emphasizes the importance of the standardization of test methods. In addition, the significant effect of the year variable shows that colistin resistance tends to increase over time.

Future studies should be conducted using standard methods and large sample sizes, and the patient and clinical characteristics of those from whom the strains were isolated should be included in publications, allowing for a more accurate assessment of the resistance rates. This increase in colistin resistance emphasizes the importance of increasing the number of studies on resistance mechanisms. The One Health approach should be adopted in the context of strictly controlling antibiotic use in hospitals and evaluating antibiotic resistance, especially in community-acquired infections, using a multidisciplinary approach.

This study had several limitations. First, the heterogeneity of the studies was high, but a meta-regression analysis was performed to understand the cause of the heterogeneity. The small sample size of some studies was one of the factors that increased the heterogeneity. In addition, the variety of tests and guidelines used to determine colistin resistance may create uncertainty in the interpretation of the results.

## 5. Conclusions

This study is one of the most comprehensive meta-analyses of colistin resistance in Turkey. Our findings reveal that colistin resistance was detected in 13.43% of all *K. pneumoniae* isolates, with significant variability depending on the testing method used. In particular, the resistance rate detected using broth microdilution was 21.58%, while the rate detected using disk diffusion was 3.87%, emphasizing the importance of standardizing susceptibility testing protocols.

A striking finding of our study is that colistin resistance has increased significantly over the years. The approximate doubling of the colistin resistance rate between 2019 and 2024 may be associated with the impact of changes in antibiotic use and infection control measures, especially after the COVID-19 pandemic. These findings indicate that infection control measures and antibiotic stewardship should be re-evaluated in the post-pandemic period.

In addition, this meta-analysis emphasizes that colistin resistance in *K. pneumoniae* has become a global health problem and that reliable diagnostic methods play a critical role, both in combating antimicrobial resistance and in determining the appropriate treatment strategies. This increase in colistin resistance poses serious challenges for healthcare systems. Due to the increase in resistant strains, the need to develop alternative treatment options and effectively implement antibiotic use policies must be emphasized. Understanding factors such as heteroresistance, biofilm formation, chromosomal mutations, plasmid-mediated resistance, efflux pump overexpression and the limitations of antibiotic resistance detection methods in Gram-negative bacteria, including *K. pneumoniae*, will allow for a more accurate assessment of the prevalence of colistin resistance, thus aiding in revising antibiotic use policies and implementing infection management. Furthermore, investigating the genetic mechanisms leading to resistance will help to develop strategies to reduce the spread of colistin-resistant strains and maintain the efficacy of last-line antibiotics.

## Data Availability

The original contributions presented in this study are included in the article/Supplementary Materials. Further inquiries can be directed to the corresponding author.

## Supplementary Materials

The following supporting information can be downloaded at: https://www.mdpi.com/article/10.3390/microorganisms13050974/s1. Table S1: Characteristics of studies included in the meta-analysis [22,23,26,27,28,29,30,31,32,33,34,35,36,37,38,39,40,41,42,43,44,45,46,47,48,49,50,51].
