# Supplementary material for: Prevalence of Colistin-Resistant Klebsiella pneumoniae Isolates in Turkey over a 20-Year Period: A Systematic Review and Meta-Analysis
Source: Microorganisms. 2025 Apr 24;13(5):974. doi: 10.3390/microorganisms13050974 (PMC12113747; doi:10.3390/microorganisms13050974)
Supplement: Supplementary file 1 [file microorganisms-13-00974-s001.zip › microorganisms-3513047-supplementary.pdf]

Table S1. Characteristics of studies included in the meta-analysis [22,23,26–51].

Study	Year period	Region	Sample	Colistin resistance confirmation method	Guideline	Total sample	Number of colistin resistant strains	Colistin resistance rate (%)
Dizbay et al., 2014 [26]	2004-2010	Central Anatolia	Different clinical samples	Disc diffusion	CLSI (2005), M100-S15	720	70	9.72
Zarakolu et al., 2016 [27]	2009-2013	Central Anatolia	Different clinical samples	Agar dilution	EUCAST (2015)	271	16	5.90
Ergonul et al., 2016 [28]	2013	Multicenter	Blood	Disc diffusion	CLSI (2010), M100-S20	221	14	6.33
Iraz et al., 2016 [29]	2012-2013	Marmara	Blood, urine, wound, sputum, TA	Gradient strip test	CLSI (2013), M100-S232	37	1	2.70
Cizmeci et al., 2017 [30]	2014-2016	Marmara	Different clinical samples	Gradient strip test	EUCAST (2014), Version 4.0 and CLSI (2015), M100–S25	69	19	27.54
Aydin et al., 2018 [31]	2014-2015	Multicenter	Blood	VITEK-2	CLSI (2016)	437	9	2.06
Ece et al., 2018 [32]	2013	Aegean Regions	TA, blood, urine, sputum	VITEK-2	-	14	0	0.00
Koyuncu Ozyurt et al., 2019 [33]	2016-2018	Mediterran ean	Different clinical samples	Colistin broth disk elution method	EUCAST (2018), Version 8.1 and CLSI	199	110	55.28
Davarci et al., 2019 [34]	2013-2014	Marmara	Blood, urine, TA, sputum	Microdilution method	CLSI (2013), M100-S232	1452	136	9.37
Arabaci et al., 2019 [22]	2017	Marmara	Blood, urine, BAL, rectal swab	Microdilution method	EUCAST (2017), Version 2.0	57	34	59.65

Ozkul Kocak and Hazirolan, 2019 [35]	2018	Central Anatolia	Blood, urine, sputum, BOS and others	Broth microdilution method	EUCAST	81	32	39.51
Kansak et al., 2020 [36]	-	Marmara	Blood, urine, wound, TA	CHROMID® Colistin R agar (COLR) (biomerieux, France)	EUCAST (2020), Version 10.0	94	61	64.89
Ozkaya et al., 2020 [23]	2016-2018	Black Sea	Different clinical samples	Disc diffusion	EUCAST (2018), Version 8.1	3334	19	0.57
Aygar., 2020 [37]	2015-2017	Central Anatolia	Blood, urine, TA, intravenous catheter	Broth microdilution method	EUCAST	66	24	36.36
Kilic et al., 2020 [38]	-	Marmara	Rectal swab, blood, urine, wound	Broth microdilution method	EUCAST	38	26	68.42
Colak et al., 2021 [39]	2019-2021	Black Sea	ETA, sputum, BAL	BD/Phoenix-100 (Becton Dickinson, ABD) automatic system	EUCAST (2021), Version 6.0-10.1	127	23	18.11
Kocer et al., 2021 [40]	2018-2019	Southeastern Anatolia	Blood, urine, sputum, TA, wound	Gradient strip test, Microdilution method	EUCAST (2018), Version 8.1	128	66	51.56
Genisel et al., 2021 [41]	2020	Southeastern Anatolia	Blood	Kirby Bauer Disc diffusion Test	EUCAST (2019), Version 9.0.	33	20	60.61
Eren et al., 2021 [42]	2013-2017	Central Anatolia	Blood	Kirby-Bauer disc diffusion	CLSI (2013), M100-S232	82	7	8.54
Unlu et al., 2021 [43]	2016-2018	Marmara	Blood, urine, TA, sputum and others	Kirby-Bauer disc diffusion	EUCAST (2021)	83	15	18.07
Suzuk Yildiz et al., 2021 [44]	2019	Multicenter	Wound, urine, TA, LRTS, blood	Broth microdilution method	EUCAST (2019), Version 9.0.	366	104	28.42
Ozmen et al., 2022 [45]	2017	Marmara	Stool	Gradient strip test	EUCAST (2017), Version 2.0.	39	2	5.13

Baykara et al., 2022 [46]	2018-2020	Eastern Anatolia	Blood, urine, sputum, TA, wound and others	Broth microdilution method	EUCAST (2009),Version 9.0.	115	48	41.74
Besli et al., 2022 [47]	-	Multicenter	Different clinical samples	Broth microdilution method	EUCAST (2014), Version 4.0.	196	56	28.57
Hosbul et al., 2022 [48]	2018-2021	Central Anatolia	TA, blood, wound, biopsy, sterile body samples and peripheral venous catheter	Broth microdilution method	EUCAST (2022), Version 12.0 and CLSI (2015), M07-A9	150	78	52.00
Mermutluoglu et al., 2023 [49]	2019-2022	Southeastern Anatolia	Blood, urine, RTS, wound, sterile body samples	Diagnostics MIC-COL test (Diagnostics, Slovakia)	EUCAST (2022), Version 12.0	425	164	38.59
Kose et al., 2023 [50]	2019-2020	Multicenter	Blood	Disc diffusion	EUCAST (2016)	50	30	60.00
Ibik et al., 2023 [51]	2017-2020	Black Sea	Blood, urine, sputum, TA	Microdilution method	EUCAST (2020), Version 10.0	32	13	40.63

* BAL: bronchoalveolar lavage, ETA: Endotracheal aspirate, LRTS: Lower respiratory tract samples, RTS: Respiratory tract samples, TA: Tracheal aspirate.
